# Supplementary material for: Secretin-dependent signals in the ventromedial hypothalamus regulate energy metabolism and bone homeostasis in mice
Source: Nat Commun. 2024 Feb 3;15:1030. doi: 10.1038/s41467-024-45436-3 (PMC10838336; doi:10.1038/s41467-024-45436-3)
Supplement: Supplementary file 1 — Supplementary information [file 41467_2024_45436_MOESM1_ESM.pdf]

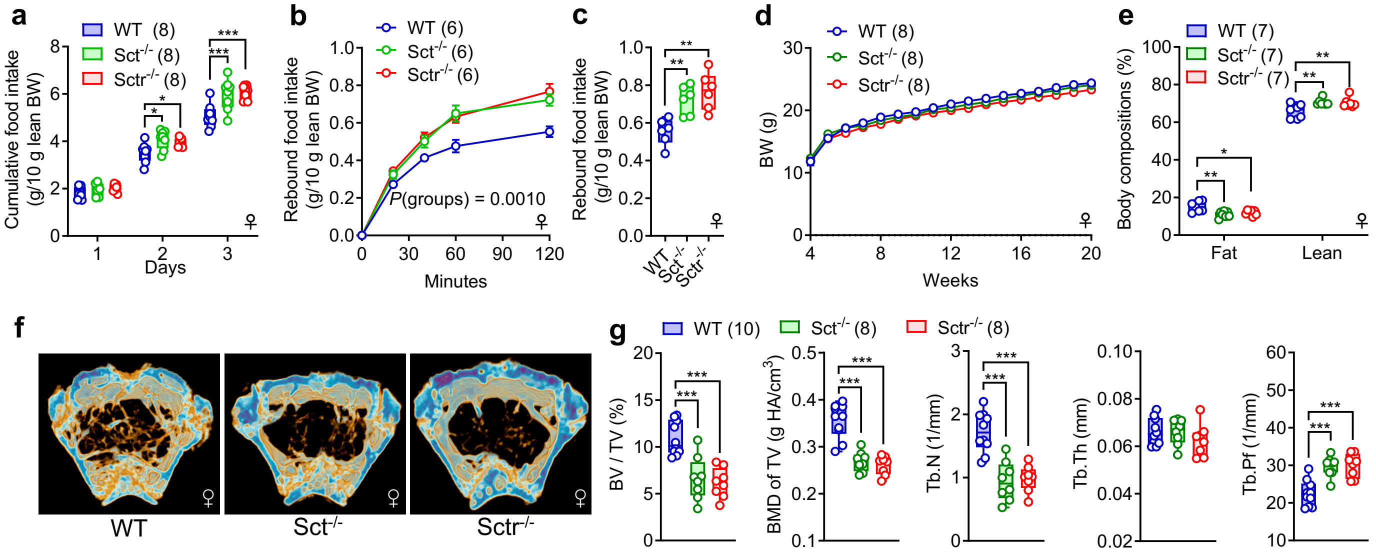

Supplementary Fig. 1. Food intake, body weight, body composition, and bone mass changes in female Sct$^{-/-}$ and Sctr$^{-/-}$ mice.

a, Cumulative daily food intake of 10-week-old female WT, Sct$^{-/-}$, and Sctr$^{-/-}$ mice in 3 consecutive days. **b**, **c**, Cumulative (**b**) and total (**c**) rebound food intake of 10-week-old female WT, Sct$^{-/-}$, and Sctr$^{-/-}$ mice after 16 hours overnight fasting. **d**, Weekly body weight changes of female WT, Sct$^{-/-}$, and Sctr$^{-/-}$ mice fed on standard rodent chow. **e**, Body composition of 18-week-old female WT, Sct$^{-/-}$, and Sctr$^{-/-}$ mice. **f**, Representative μCT images showing the reduction in trabecular bone mass in the femurs of 20-week-old female Sct$^{-/-}$ and Sctr$^{-/-}$ mice. **g**, Corresponding measurements of (**f**): BV/TV, BMD of TV, Tb.N, Tb.Th, and Tb.Pf. Numbers in parentheses in each graph indicate sample size. Box plots with whiskers from minima to maxima, the central line at the 50th percentile, and the ends of the box at the 25th and 75th percentiles. (**a**, **b**, **d**, **e**) Two-way ANOVA with Holm–Šídák multiple comparisons test. (**c**, **g**) One-way ANOVA with Holm–Šídák multiple comparisons test. **P* < 0.05; ***P* < 0.01; ****P* < 0.001 *vs* WT. Error bars represent SEM. Source data are provided as a Source Data file.

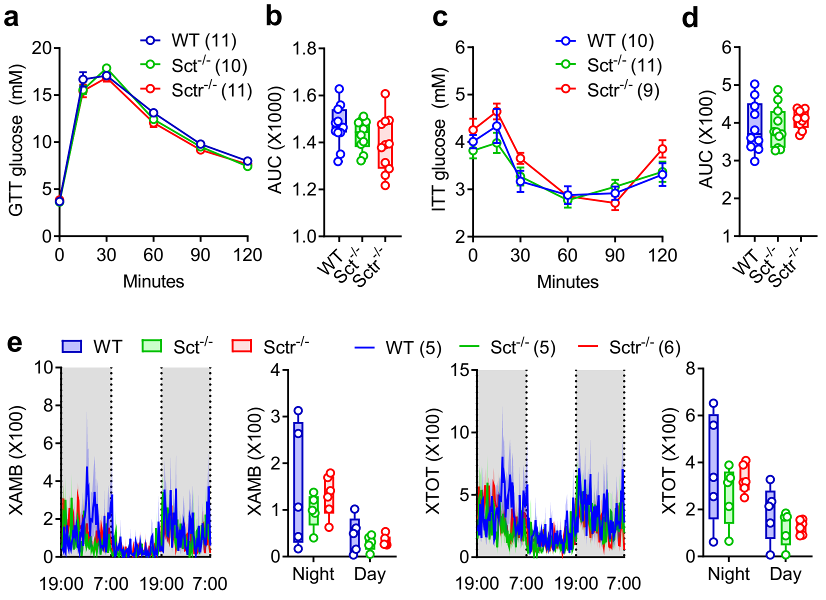

Supplementary Fig. 2. Systemic SCT or SCTR KO did not alter glucose homeostasis and motor activity. **a**, **b**, Glucose tolerance test of 16-week-old male WT, Sct$^{-/-}$, and Sctr$^{-/-}$ mice. **c**, **d**, Insulin tolerance test of 16-week-old male WT, Sct$^{-/-}$, and Sctr$^{-/-}$ mice. **e**, Temporal changes of motor activity in 16-week-old male WT, Sct$^{-/-}$, and Sctr$^{-/-}$ mice. AUC, area under the curve. XAMB, ambulatory activity count. XTOT, total horizontal motor activity. Numbers in parentheses in each graph indicate sample size. Box plots with whiskers from minima to maxima, the central line at the 50th percentile, and the ends of the box at the 25th and 75th percentiles. (**a**, **c**, **e**) Two-way ANOVA with Holm–Šídák multiple comparisons test. (**b**, **d**) One-way ANOVA with Holm–Šídák multiple comparisons test. Error bars represent SEM. Source data are provided as a Source Data file.

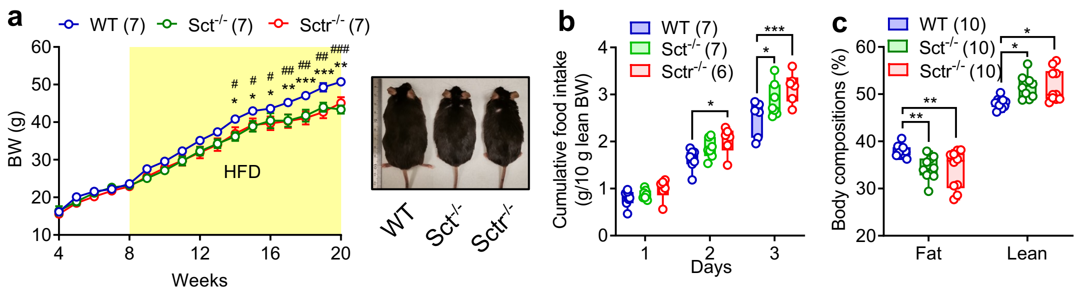

Supplementary Fig. 3. Body weight, food intake, and body composition of male WT, Sct$^{-/-}$, and Sctr$^{-/-}$ mice fed on HFD.

a, Left: weekly body weight changes of HFD-fed male WT, Sct$^{-/-}$, and Sctr$^{-/-}$ mice. The yellow shaded area indicates that the mice were fed HFD. "#" and "*" represent the comparison results of WT *vs* Sct$^{-/-}$ and WT *vs* Sctr$^{-/-}$, respectively. Right: representative photographs of 20-week-old male mice. **b**, Cumulative daily food intake of 16-week-old HFD-fed male WT, Sct$^{-/-}$, and Sctr$^{-/-}$ mice in 3 consecutive days. **c**, Body composition of 18-week-old HFD-fed male WT, Sct$^{-/-}$, and Sctr$^{-/-}$ mice. Numbers in parentheses in each graph indicate sample size. Box plots with whiskers from minima to maxima, the central line at the 50th percentile, and the ends of the box at the 25th and 75th percentiles. Two-way ANOVA with Holm–Šídák multiple comparisons test. $^{\#/*}P < 0.05$; $^{\#\#/**}P < 0.01$; $^{\#\#\#/***}P < 0.001$. Error bars represent SEM. Source data are provided as a Source Data file.

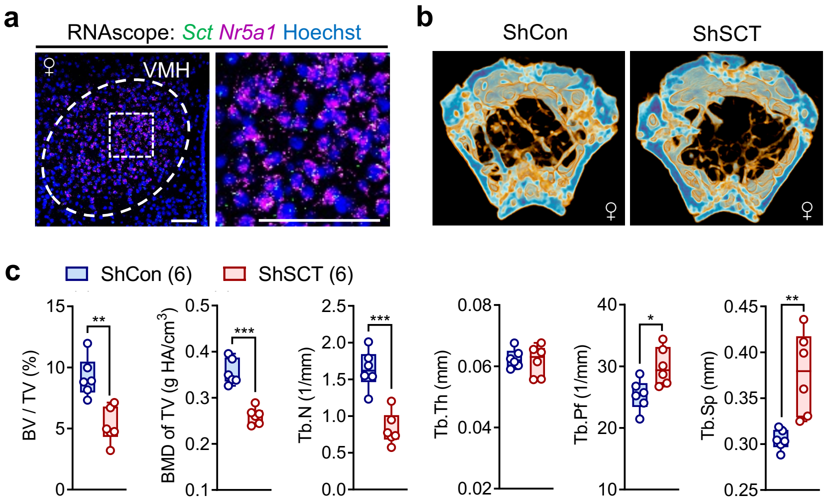

Supplementary Fig. 4. VMH-specific SCT KD decreases bone mass in female mice. **a**, RNAscope *in situ* hybridization of *Sct* and *Nr5a1* in VMH of female mouse. Scale bars = 50 μm. **b**, Representative μCT images of femurs from 20-week-old female ShSCT and ShCon littermates. **c**, Corresponding measurements of (**b**): BV/TV, BMD of TV, Tb.N, Tb.Th, Tb.Pf, and Tb.Sp. Numbers in parentheses in each graph indicate sample size. Box plots with whiskers from minima to maxima, the central line at the 50th percentile, and the ends of the box at the 25th and 75th percentiles. Two-tailed Student's *t*-test. $*P < 0.05$; $**P < 0.01$. Error bars represent SEM. Source data are provided as a Source Data file.

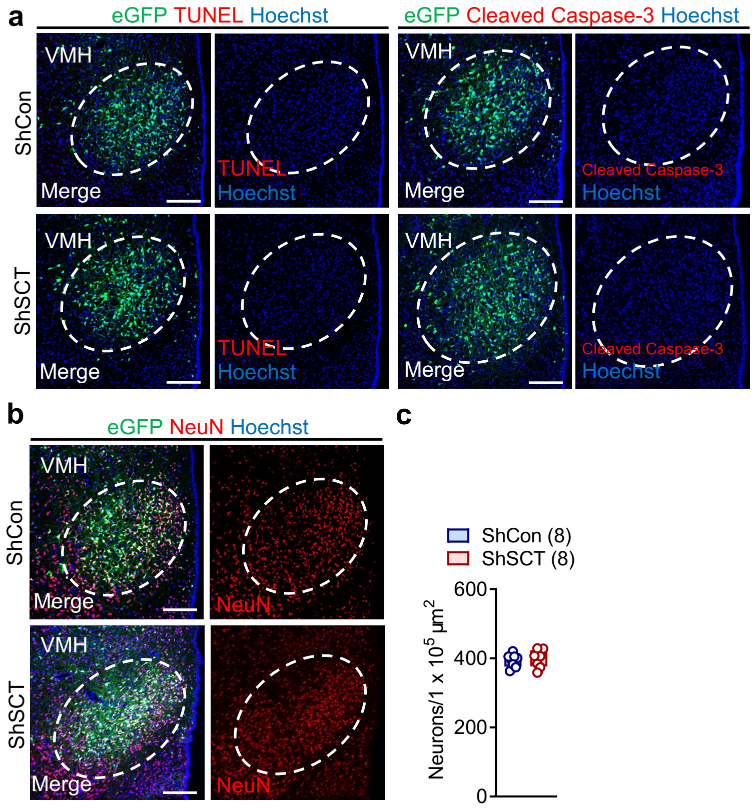

Supplementary Fig. 5. VMH-specific SCT KD did not affect cell survival and neuronal density in the VMH.

a, Immunofluorescence staining of apoptosis markers (TUNEL and Cleaved Caspase-3) in VMH of ShSCT and ShCon littermates. **b**, Immunofluorescence staining of NeuN in VMH of ShSCT and ShCon mice. **c**, Neuronal density calculated based on NeuN staining (**b**). eGFP indicates the area of virus injection. Numbers in parentheses in each graph indicate sample size. Box plots with whiskers from minima to maxima, the central line at the 50th percentile, and the ends of the box at the 25th and 75th percentiles. Two-tailed Student's *t*-test. Error bars represent SEM. Scale bar = 100 μm. Source data are provided as a Source Data file.

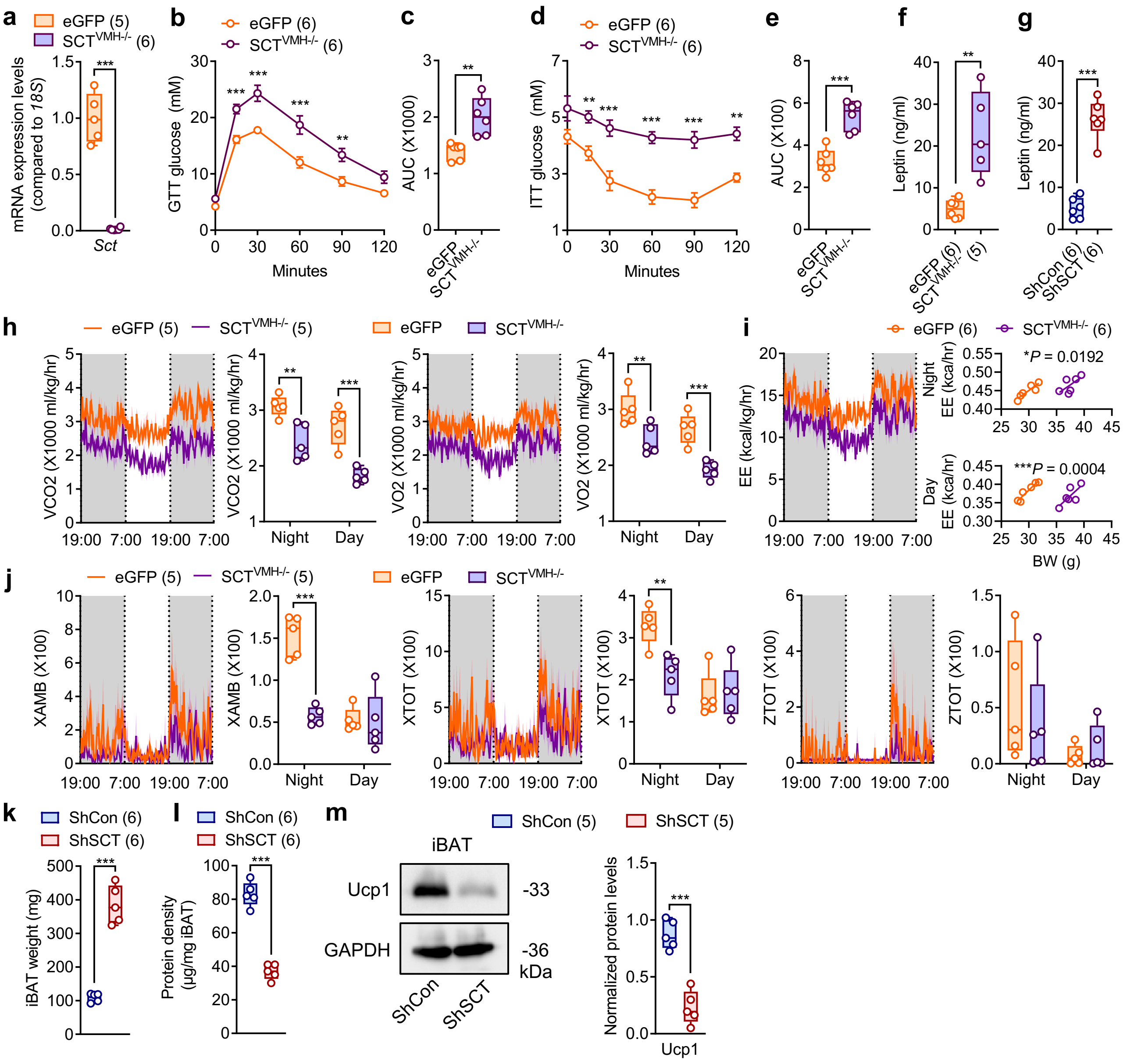

Supplementary Fig. 6. VMH-specific SCT KD induces obesity-related phenotypes.

a, Reduced transcript levels of *Sct* in VMH after Cre-mediated KD. **b**, **c**, Glucose tolerance test of $SCT^{VMH-/-}$ and eGFP littermates. **d**, **e**, Insulin tolerance test of $SCT^{VMH-/-}$ and eGFP littermates. **f**, Serum leptin levels in $SCT^{VMH-/-}$ and eGFP littermates. **g**, Serum leptin levels in ShSCT and ShCon littermates. **h**, Temporal changes of VCO2 and VO2 in $SCT^{VMH-/-}$ and eGFP littermates. **i**, Temporal changes of EE in $SCT^{VMH-/-}$ and eGFP littermates. **j**, Temporal changes of motor activity in $SCT^{VMH-/-}$ and eGFP littermates. **k**, Tissue weight of iBAT. **l**, Protein density of iBAT. **m**, Western blot of Ucp1 in iBAT. AUC, area under the curve. XAMB, ambulatory activity count. XTOT, total horizontal motor activity. ZTOT, total vertical motor activity. Numbers in parentheses in each graph indicate sample size. Box plots with whiskers from minima to maxima, the central line at the 50th percentile, and the ends of the box at the 25th and 75th percentiles. (**a**, **c**, **e**, **f**, **g**, **k**, **l**, **m**) Two-tailed Student's *t*-test. (**b**, **d**, **h**, **j**) Two-way ANOVA with Holm–Šídák multiple comparisons test. (**i**) One-way ANCOVA with pairwise comparisons on adjusted means. $*P < 0.05$; $**P < 0.01$; $***P < 0.001$. Error bars represent SEM. Source data are provided as a Source Data file.

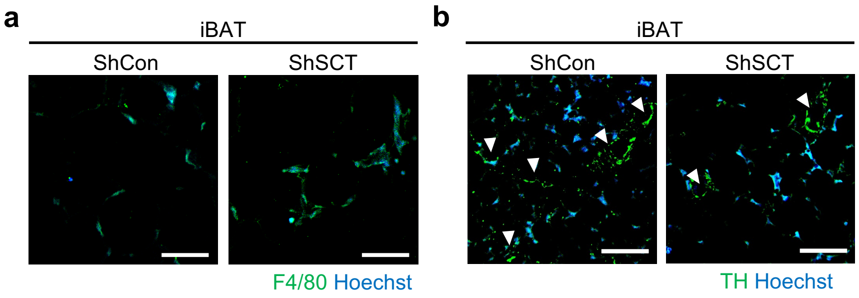

Supplementary Fig. 7. VMH-specific SCT KD resulted in chronic inflammation and impaired sympathetic innervation in iBAT.
a, Representative immunofluorescent images showing the presence of F4/80-positive macrophages in iBAT. **b**, Representative immunofluorescent images showing the presence of TH-positive sympathetic nerves in iBAT. Arrows indicate TH-positive signals. Scale bar = 500 µm.

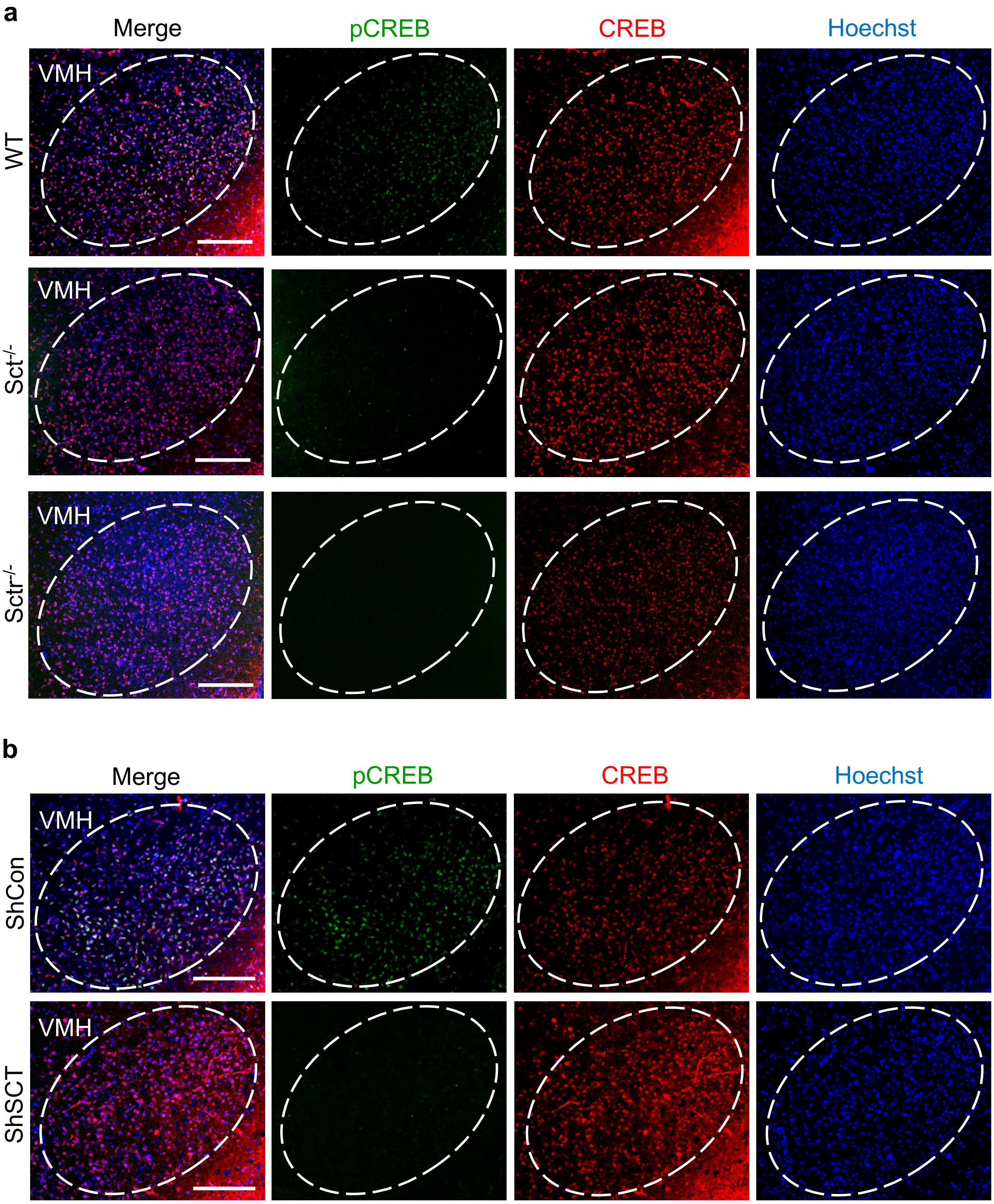

Supplementary Fig. 8. Systemic SCT or SCTR KO and VMH-specific SCT KD decreases the pCREB levels in the VMH.

a, Representative immunofluorescent images showing the phosphorylation of CREB in VMH of 20-week-old male WT, $Sct^{-/-}$, and $Sctr^{-/-}$ mice. **b**, Representative immunofluorescent images showing the phosphorylation of CREB in VMH of 20-week-old male ShSCT and ShCon littermates. Scale bar = 100 μm.

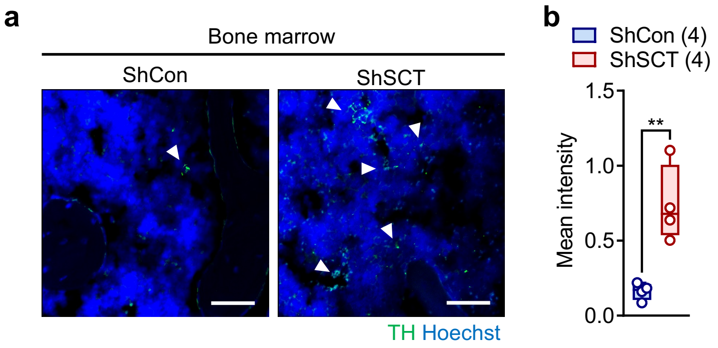

Supplementary Fig. 9. VMH-specific SCT KD enhances sympathetic output in bone tissue.
a, Representative immunofluorescent images showing the presence of TH positive sympathetic nerves in bone marrow of ShSCT and ShCon littermates. Arrows indicate TH-positive signals. Scale bar = 500 μm. **b**, Quantitative analysis of (**a**). Numbers in parentheses in each graph indicate sample size. Box plots with whiskers from minima to maxima, the central line at the 50th percentile, and the ends of the box at the 25th and 75th percentiles. Two-tailed Student's *t*-test. ***P* < 0.01. Error bars represent SEM. Source data are provided as a Source Data file.

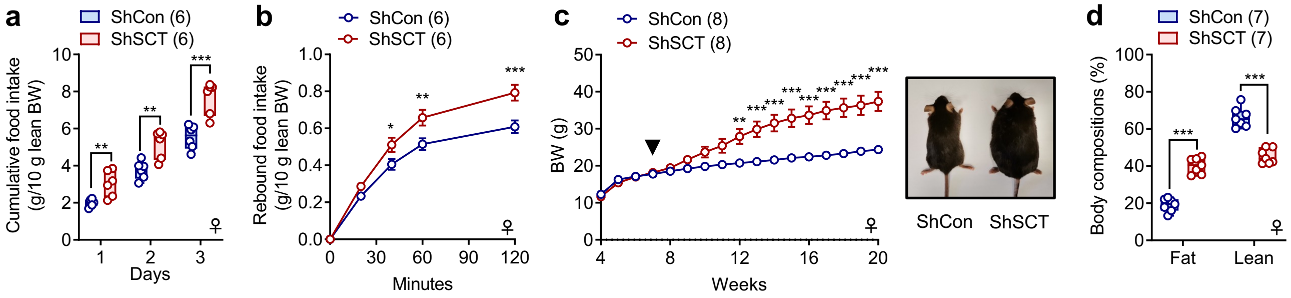

Supplementary Fig. 10. VMH-specific SCT KD leads to hyperphagia and obesity in female mice. **a**, Daily food intake of 10-week-old female ShSCT and ShCon littermates in 3 consecutive days. **b**, Rebound food intake of 10-week-old overnight fasted (16 hours) female ShSCT and ShCon littermates. **c**, Left: weekly body weight changes of female ShSCT and ShCon littermates fed on standard rodent chow. Black arrow indicates virus injection at 7 weeks of age. Right: representative photographs of 20-week-old mice. **d**, Body composition of 18-week-old female ShSCT and ShCon littermates. Numbers in parentheses in each graph indicate sample size. Box plots with whiskers from minima to maxima, the central line at the 50th percentile, and the ends of the box at the 25th and 75th percentiles. Two-way ANOVA with Holm–Šídák multiple comparisons test. *$P < 0.05$; **$P < 0.01$; ***$P < 0.001$. Error bars represent SEM. Source data are provided as a Source Data file.

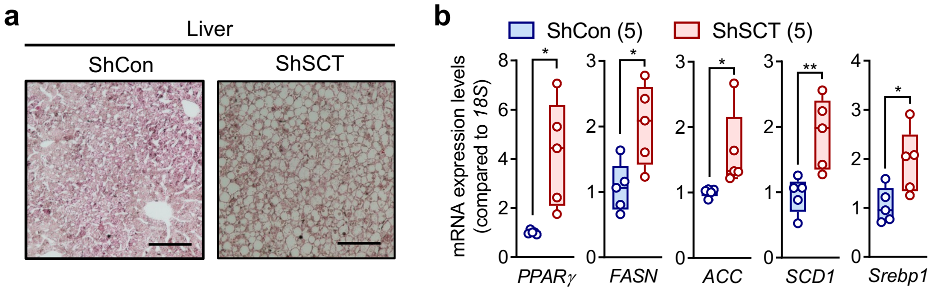

Supplementary Fig. 11. VMH-specific SCT KD enhances lipogenesis.
a, H&E staining of liver from male ShSCT and ShCon littermates. Scale bar = 100 um. **b**, Relative expression of lipogenesis-related genes in the liver. Numbers in parentheses in each graph indicate sample size. Box plots with whiskers from minima to maxima, the central line at the 50th percentile, and the ends of the box at the 25th and 75th percentiles. Two-tailed Student's *t*-test. *$P < 0.05$; **$P < 0.01$. Error bars represent SEM. Source data are provided as a Source Data file.

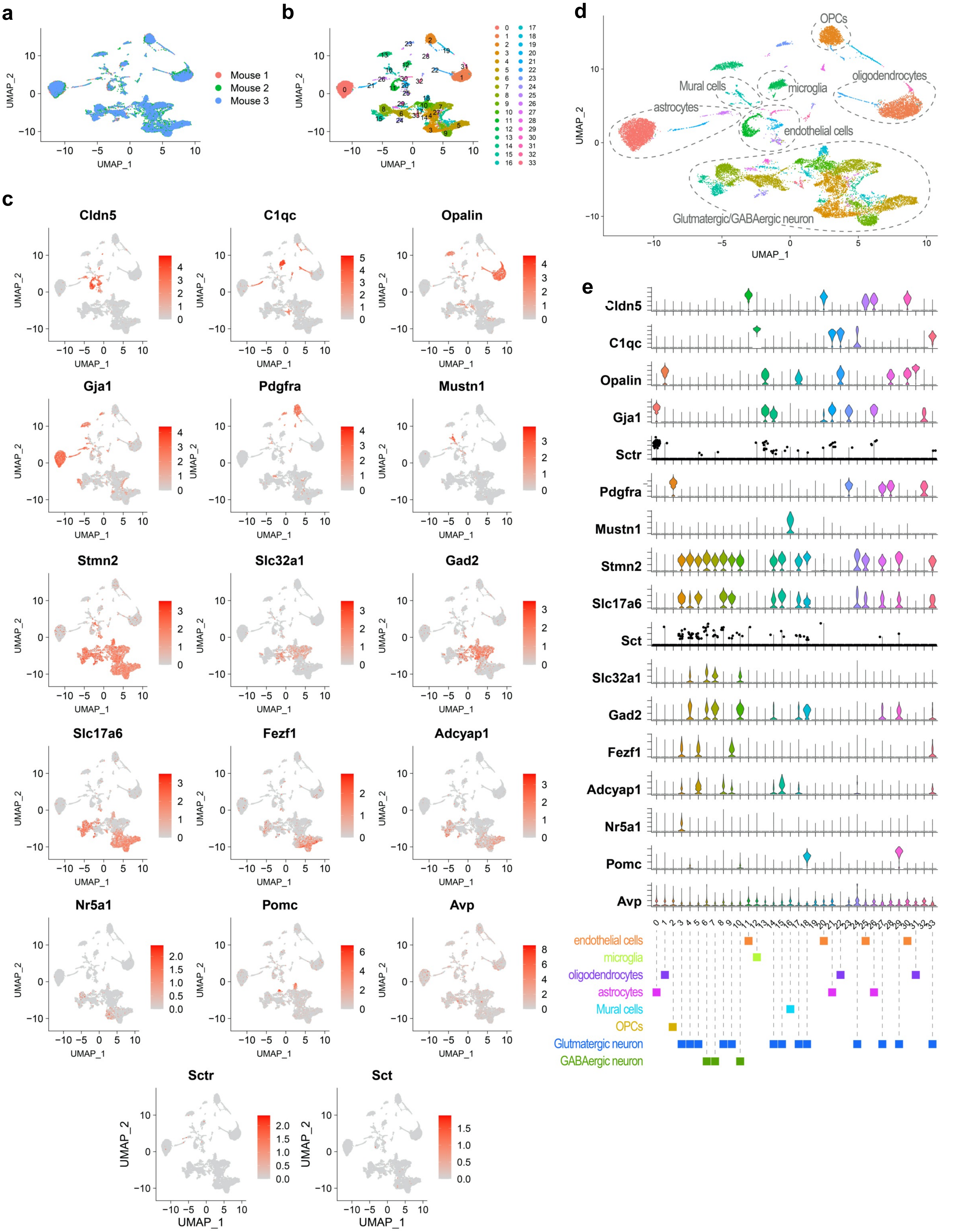

Supplementary Fig. 12. A reanalysis of single-cell sequencing data confirmed the expression of *Sctr* and *Sct* in VMH. **a**,**b**, UMAP plots showing the 33 clusters of VMH cells integrated from three mice. **c**, The expressions of *Sctr*, *Sct*, and marker genes for endothelial cells (*Cldn5*), microglia (*C1qc*), oligodendrocytes (*Opalin*), astrocytes (*Gja1*), oligodendrocyte progenitor cells (*Pdgfra*), mural cells (*Mustn1*), neurons (*Stmn2*), glutamatergic neurons (*Slc17a6*, *Fezf1*, and *Adcyap1*), and GABAergic neurons (*Slc32a1* and *Gad2*) are color coded (Red) on UMAP plots. **d**, Both non-neuronal and neuronal clusters are outlined and labelled in UMAP plot. **e**, Violin plots show differential expressions of marker genes, as well as *Sct* and *Sctr* for all 33 clusters. Raw data were obtained from Mendeley Data (https://doi.org/10.17632/ypx3sw2f7c.1). Detailed information for these data can be found in the following paper "Dong-Wook Kim. Multimodal Analysis of Cell Types in a Hypothalamic Node Controlling Social Behavior. *Cell* 179.3 (2019)".

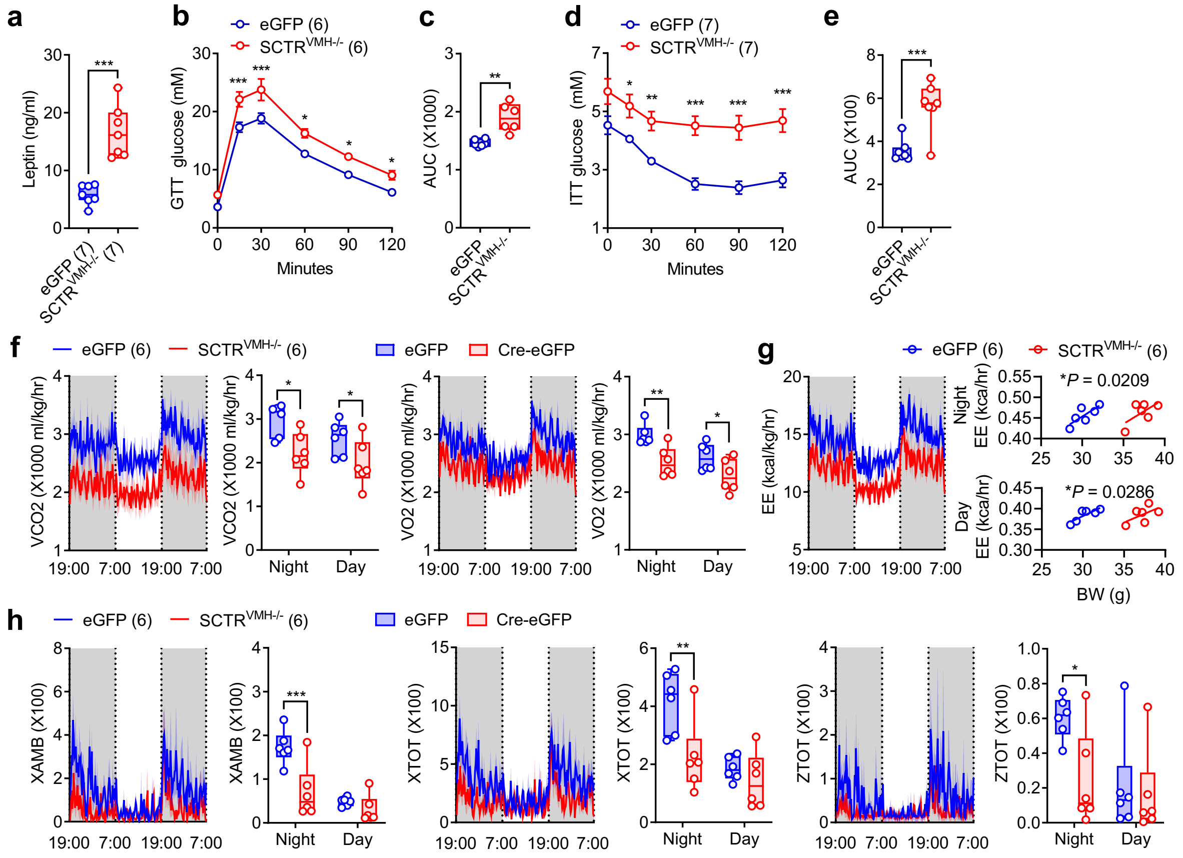

Supplementary Fig. 13. Cre-mediated SCTR KD in VMH causes metabolic dysfunction.
a, Serum leptin levels in 20-week-old SCTR$^{VMH-/-}$ and eGFP littermates. **b**, **c**, Glucose tolerance test of 16-week-old SCTR$^{VMH-/-}$ and eGFP littermates. **d**, **e**, Insulin tolerance test of 16-week-old SCTR$^{VMH-/-}$ and eGFP littermates. **f**, Temporal changes of VO2 and VCO2 in 16-week-old SCTR$^{VMH-/-}$ and eGFP littermates. **g**, Temporal changes of EE in 16-week-old SCTR$^{VMH-/-}$ and eGFP littermates. **h**, Temporal changes of motor activity in 16-week-old SCTR$^{VMH-/-}$ and eGFP littermates. AUC, area under the curve. XAMB, ambulatory activity count. XTOT, total horizontal motor activity. ZTOT, total vertical motor activity. Numbers in parentheses in each graph indicate sample size. Box plots with whiskers from minima to maxima, the central line at the 50th percentile, and the ends of the box at the 25th and 75th percentiles. (**a**, **c**, **e**) Two-tailed Student's *t*-test. (**b**, **d**, **f**, **h**) Two-way ANOVA with Holm–Šídák multiple comparisons test. (**g**) One-way ANCOVA with pairwise comparisons on adjusted means. $*P < 0.05$; $**P < 0.01$; $***P < 0.001$. Error bars represent SEM. Source data are provided as a Source Data file.

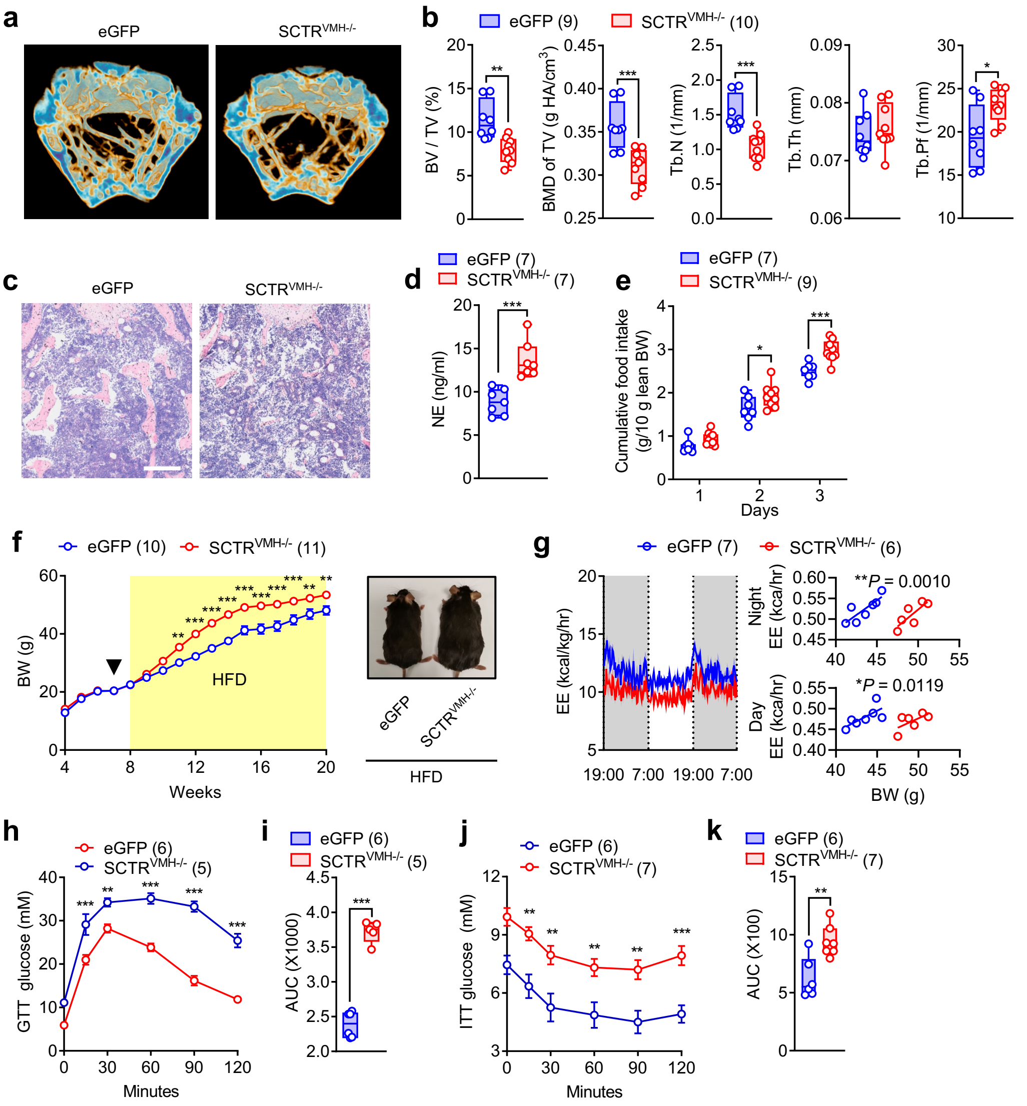

Supplementary Fig. 14. Cre-mediated SCTR KD in VMH exacerbates osteopenia and obesity in DIO mice. **a**, Representative μCT images of femurs from 20-week-old HFD-fed SCTR$^{VMH-/-}$ and eGFP littermates. **b**, Corresponding measurements of (**a**): BV/TV, BMD of TV, Tb.N, Tb.Th, and Tb.Pf. **c**, Representative femoral H&E staining images of 20-week-old HFD-fed SCTR$^{VMH-/-}$ and eGFP littermates. Scale bar = 250 μm. **d**, Serum NE levels in HFD-fed SCTR$^{VMH-/-}$ and eGFP littermates. **e**, Cumulative daily food intake of 10-week-old HFD-fed SCTR$^{VMH-/-}$ and eGFP littermates in 3 consecutive days. **f**, Left: weekly body weight changes of SCTR$^{VMH-/-}$ and eGFP littermates fed on HFD. Black arrow indicates virus injection at 7 weeks of age. The yellow shaded area indicates that the mice were fed HFD. Right: representative photographs of 20-week-old mice. **g**, Temporal changes of EE in 16-week-old HFD-fed SCTR$^{VMH-/-}$ and eGFP littermates. **h**, **i**, Glucose tolerance test of 16-week-old HFD-fed SCTR$^{VMH-/-}$ and eGFP littermates. **j**, **k**, Insulin tolerance test of 16-week-old HFD-fed SCTR$^{VMH-/-}$ and eGFP littermates. AUC, area under the curve. Numbers in parentheses in each graph indicate sample size. Box plots with whiskers from minima to maxima, the central line at the 50th percentile, and the ends of the box at the 25th and 75th percentiles. (**b, d, i, k**) Two-tailed Student's *t*-test. (**e**, **f**, **h**, **j**) Two-way ANOVA with Holm–Šídák multiple comparisons test. (**g**) One-way ANCOVA with pairwise comparisons on adjusted means. $^{*}P < 0.05$; $^{**}P < 0.01$; $^{***}P < 0.001$. Error bars represent SEM. Source data are provided as a Source Data file.

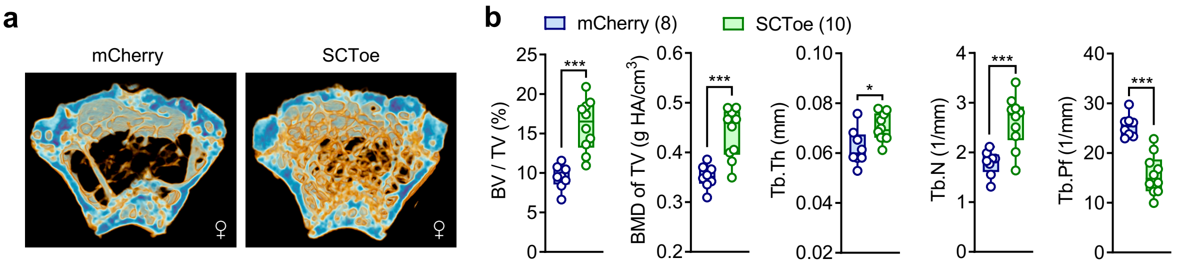

Supplementary Fig. 15. SCT overexpression in the VMH of female mice increases bone mass. **a**, Representative μCT images of femurs from 20-week-old female SCToe and mCherry littermates. **b**, Corresponding measurements of (**a**): BV/TV, BMD of TV, Tb.Th., Tb.N, and Tb.Pf. Numbers in parentheses in each graph indicate sample size. Box plots with whiskers from minima to maxima, the central line at the 50th percentile, and the ends of the box at the 25th and 75th percentiles. Two-tailed Student's *t*-test. **P* < 0.05; ****P* < 0.001. Error bars represent SEM. Source data are provided as a Source Data file.

Supplementary Table 1. Primer sequences.

Gene name	Forward	Reverse
18S	CTCTAGATAACCTCGGGCC	GAACCCTGATTCCCCGTCA
Sct	GACCATGGAGCCTCCGCTG	GGACAACCAATCCCTACTCC
Sctr	GGTGGAGGGCCTCTATCTTC	CCAGGCGCTTATAATGGTGT
COX2	ATAACCGAGTCGTTCTGCCAAT	TTTCAGAGCATTGGCCATAGAA
Rps18	TGTGTTAGGGGACTGGTGGACA	CATCACCCACTTACCCCCAAAA
PPARγ	TCAGCTCTGTGGACCTCTCC	ACCCTTGCATCCTTCACAAG
FASN	GGTGTGGTGGGTTTGGTGAATTGT	TCACGAGGTCATGCTTTAGCACCT
ACC	GACAGACTGATCGCAGAGAAAG	TGGAGAGCCCCACACACA
Cox7a1	GCTCTGGTCCGGTCTTTTAG	CTTTCAAGTGTACTGGGAGGTC
Cox8b	GAACCATGAAGCCAACGACT	GCGAAGTTCACAGTGGTTCC
Ucp1	GTACACCAAGGAAGGACCGA	TTTATTCGTGGTCTCCCAGC
Pgc1α	AACCACACCCACAGGATCAGA	AGGAGGCAAAGGATGATTCG
Dio2	TGTCTGGAACAGCTTCCTCC	AGTGAAAGGTGGTCAGGTGG
Tfam	AGGAGGCAAAGGATGATTCG	CCTCAGGAGACAGATTTTTCCA
Srebp1	GGAGCCATGGATTGCACATT	GGCCCGGGAAGTCACTGT
CIDEA	CATACATGCTCCGAGTACTGG	CATCCCACAGCCTATAACAGAG
FGF21	CAAATCCTGGGTGTCAAAGC	CATGGGCTTCAGACTGGTAC
POMC	CCCTCCTGCTTCAGACCTC	CGTTGCCAGGAAACACGG
AgRP	GTCTAAGTCTGAATGGCCTCAAG	CATCCATTGGCTAGGTGCGAC
NPY	CTGACCCTCGCTCTATCTCTGC	CCATCACCACATGGAAGGGTCT
SCD1	CCGGAGACCCTTAGATCGA	TAGCCTGTAAAAGATTTCTGCAAACC
F4/80	CTTTGGCTATGGGCTTCCAGTC	GCAAGGAGGACAGAGTTTATCGTG
MCP-1	CCCAATGAGTAGGCTGGAGA	TCTGGACCCATTCCTTCTTG
CD11c	CTGGATAGCCTTTCTTCTGCTG	GCACACTGTGTCCGAACTCA
TH	CCAAGGTTCATTGGACGGC	CTCTCCTCGAATACCACAGC
Adrb1	GGAGCTCCCTCGGACGAC	AGCCTGGCTCTCTACACCTTG
Adrb2	GTACTGTGCCTAGCCTTAGCGT	GGTTAGTGTCCTGTCAAGGAGG
Adrb3	CCGTGAAGATCCAGCAAGGA	GGTTCTGGAGCGTTGGAGAGT
