# Supplementary material for: Secretin-dependent signals in the ventromedial hypothalamus regulate energy metabolism and bone homeostasis in mice
Source: Nat Commun. 2024 Feb 3;15:1030. doi: 10.1038/s41467-024-45436-3 (PMC10838336; doi:10.1038/s41467-024-45436-3)
Supplement: Supplementary file 3 — Reporting Summary [file 41467_2024_45436_MOESM3_ESM.pdf]

nature portfolio

Corresponding author(s): Billy Kwok Chong Chow
Wei Qiao
Kelvin Wai-Kwok Yeung

Last updated by author(s): Jan 11, 2024

Reporting Summary

Nature Portfolio wishes to improve the reproducibility of the work that we publish. This form provides structure for consistency and transparency in reporting. For further information on Nature Portfolio policies, see our Editorial Policies and the Editorial Policy Checklist.

Statistics

For all statistical analyses, confirm that the following items are present in the figure legend, table legend, main text, or Methods section.

n/a	Confirmed	
☐	☒	The exact sample size (*n*) for each experimental group/condition, given as a discrete number and unit of measurement
☐	☒	A statement on whether measurements were taken from distinct samples or whether the same sample was measured repeatedly
☐	☒	The statistical test(s) used AND whether they are one- or two-sided<br>*Only common tests should be described solely by name; describe more complex techniques in the Methods section.*
☐	☒	A description of all covariates tested
☐	☒	A description of any assumptions or corrections, such as tests of normality and adjustment for multiple comparisons
☐	☒	A full description of the statistical parameters including central tendency (e.g. means) or other basic estimates (e.g. regression coefficient) AND variation (e.g. standard deviation) or associated estimates of uncertainty (e.g. confidence intervals)
☐	☒	For null hypothesis testing, the test statistic (e.g. *F*, *t*, *r*) with confidence intervals, effect sizes, degrees of freedom and *P* value noted<br>*Give P values as exact values whenever suitable.*
☒	☐	For Bayesian analysis, information on the choice of priors and Markov chain Monte Carlo settings
☒	☐	For hierarchical and complex designs, identification of the appropriate level for tests and full reporting of outcomes
☒	☐	Estimates of effect sizes (e.g. Cohen's *d*, Pearson's *r*), indicating how they were calculated

Our web collection on statistics for biologists contains articles on many of the points above.

Software and code

Policy information about availability of computer code

Data collection	Oxymax for Windows (v 5.0)
Data analysis	Prism (v 7.0), GraphPad, https://www.graphpad.com/scientific software/prism/<br>ImageJ (v 1.54h), National Institutes of Health https://imagej.nih.gov/ij/<br>CTAn (v 1.0), Bruker, https://blue-scientific.com/bruker-ctan-micro-ct-software/<br>NRecon (v 2.0), Bruker, https://www.microphotonics.com/micro-ct-systems/nrecon reconstruction-software/<br>CTvox/CTvol (v 1.0), Bruker, https://blue-scientific.com/ctvox-micro-ct-volume-rendering-software/<br>Seurat (v 4.2.0), Satija lab, https://satijalab.org/seurat/.<br>R (4.2.2) find code: GitHub (https://github.com/nedchen2/HKFI-Secretin-Signaling/) or Zenodo (https://doi.org/10.5281/zenodo.10476920).)

For manuscripts utilizing custom algorithms or software that are central to the research but not yet described in published literature, software must be made available to editors and reviewers. We strongly encourage code deposition in a community repository (e.g. GitHub). See the Nature Portfolio guidelines for submitting code & software for further information.

Data

Policy information about availability of data

All manuscripts must include a data availability statement. This statement should provide the following information, where applicable:

- Accession codes, unique identifiers, or web links for publicly available datasets
- A description of any restrictions on data availability
- For clinical datasets or third party data, please ensure that the statement adheres to our policy

Find "Single-cell RNA-seq Data: Kim et al., 2019" in Mendeley Data https://doi.org/10.17632/ypx3sw2f7c.1
All data generated or analysed during this study are included in this article (and its supplementary information files). Source data are provided with this paper.

Research involving human participants, their data, or biological material

Policy information about studies with human participants or human data. See also policy information about sex, gender (identity/presentation), and sexual orientation and race, ethnicity and racism.

Reporting on sex and gender	N/A
Reporting on race, ethnicity, or other socially relevant groupings	N/A
Population characteristics	N/A
Recruitment	N/A
Ethics oversight	N/A

Note that full information on the approval of the study protocol must also be provided in the manuscript.

Field-specific reporting

Please select the one below that is the best fit for your research. If you are not sure, read the appropriate sections before making your selection.

☒ Life sciences ☐ Behavioural & social sciences ☐ Ecological, evolutionary & environmental sciences

For a reference copy of the document with all sections, see nature.com/documents/nr-reporting-summary-flat.pdf

Life sciences study design

All studies must disclose on these points even when the disclosure is negative.

Sample size	Sample sizes were determined based on comparable experiments and the Power & Animal Number Calculator (https://ccmr.hku.hk/en/Training/Experimental-Design)
Data exclusions	No data was excluded.
Replication	Experiments in this study were repeated independently at least three times and all replications were successful.
Randomization	All animals used were aged 4 weeks and litter mates were randomly assigned to experimental groups.<br>For studies involving body weight and body composition, body weight was measured at the beginning of the experiments and randomized to avoid significant difference in body weight among the different experimental groups.<br>All metabolic data are assigned a random order.
Blinding	The investigator in data collection and processing was blinded.

Reporting for specific materials, systems and methods

We require information from authors about some types of materials, experimental systems and methods used in many studies. Here, indicate whether each material, system or method listed is relevant to your study. If you are not sure if a list item applies to your research, read the appropriate section before selecting a response.

Materials & experimental systems

n/a	Involved in the study
☐	☒ Antibodies
☒	☐ Eukaryotic cell lines
☒	☐ Palaeontology and archaeology
☐	☒ Animals and other organisms
☒	☐ Clinical data
☒	☐ Dual use research of concern
☒	☐ Plants

Methods

n/a	Involved in the study
☒	☐ ChIP-seq
☒	☐ Flow cytometry
☒	☐ MRI-based neuroimaging

Antibodies

Antibodies used

Rabbit anti-SCTR, Sigma-Aldrich, Cat# HPA007269; RRID:AB_1856640
Rabbit anti-SCT, Phoenix Pharmaceuticals, Cat# G-067-04; RRID:AB_2650428
Rabbit anti-NeuN, Millipore, Cat# ABN78 ; RRID:AB_10807945
Rabbit anti-Cleaved Caspase-3 (Asp175), Cell Signalling Technology, Cat# 9661; RRID:AB_2341188
Rabbit anti-pCREB, Abcam, Cat# ab32096; RRID:AB_731734
Mouse anti-CREB, Abcam, Cat# ab178322; RRID:AB_2827810
Chicken anti-GFAP, Abcam, Cat# ab4674; RRID:AB_304558
rabbit anti-F4/80, Cell Signalling Technology, Cat# 70076; RRID:AB_2799771
Rabbit anti-TH, Millipore, Cat# AB152; RRID:AB_390204
rabbit anti-Ucp1, Sigma-Aldrich, Cat# U6382;RRID:AB_261838
Rabbit anti-GAPDH, Cell Signalling Technology, Cat# 2118; RRID:AB_561053
AlexaFluor 594 donkey anti-mouse, Invitrogen, Cat# A21203; RRID:AB_141633
DyLight 680 goat anti-chicken, Invitrogen, Cat# SA5-10074; RRID:AB_2556654
AlexaFluor 488 donkey anti-rabbit, Invitrogen, Cat# A32790; RRID:AB_2762833
Peroxidase-conjugated anti-rabbit,Cell Signalling Technology, Cat# 7074; RRID:AB_2099233
Peroxidase-conjugated anti-mouse, Invitrogen, Cat# 31430; RRID:AB_228307

Validation

Rabbit anti-SCTR, Sigma-Aldrich, Cat# HPA007269; https://www.cell.com/cell/fulltext/S0092-8674(18)31324-2?_returnURL=https%3A%2F%2Flinkinghub.elsevier.com%2Fretrieve%2Fpii%2FS0092867418313242%3Fshowall%3Dtrue
Rabbit anti-SCT, Phoenix Pharmaceuticals, Cat# G-067-04; https://www.cell.com/cell/fulltext/S0092-8674(18)31324-2?_returnURL=https%3A%2F%2Flinkinghub.elsevier.com%2Fretrieve%2Fpii%2FS0092867418313242%3Fshowall%3Dtrue
Rabbit anti-NeuN, Millipore, Cat# ABN78 ; https://www.sigmaaldrich.com/HK/zh/product/mm/abn78?utm_source=google&utm_medium=cpc&utm_campaign=19475204102&utm_content=143558715063&gclid=Cj0KCQiAnfmsBhDfARIsAM7MKi2_5rmLkDvK1AfgxXHT_uyhF3h4mjtmUu_0v7kXzdQI9_SJbGUSU1IaApFBEALw_wcB
Rabbit anti-Cleaved Caspase-3 (Asp175), Cell Signalling Technology, Cat# 9661; https://www.cellsignal.com/products/primary-antibodies/cleaved-caspase-3-asp175-antibody/9661
Rabbit anti-pCREB, Abcam, Cat# ab32096; https://www.abcam.com/products/primary-antibodies/creb-phospho-s133-antibody-e113-ab32096.html
Mouse anti-CREB, Abcam, Cat# ab178322; https://www.abcam.com/products/primary-antibodies/creb-antibody-lb9-ab178322.html
Chicken anti-GFAP, Abcam, Cat# ab4674; https://www.abcam.com/products/primary-antibodies/gfap-antibody-ab4674.html
rabbit anti-F4/80, Cell Signalling Technology, Cat# 70076; https://www.cellsignal.com/products/primary-antibodies/f4-80-d2s9r-xp-rabbit-mab/70076
Rabbit anti-TH, Millipore, Cat# AB152; https://www.merckmillipore.com/HK/en/product/Anti-Tyrosine-Hydroxylase-Antibody,MM_NF-AB152?ReferrerURL=https%3A%2F%2Fwww.google.com%2F
rabbit anti-Ucp1, Sigma-Aldrich, Cat# U6382;https://www.sigmaaldrich.com/HK/zh/product/sigma/u6382
Rabbit anti-GAPDH, Cell Signalling Technology, Cat# 2118; https://www.cellsignal.com/product/productDetail.jsp?productId=2118&utm_medium=b2b&utm_campaign=general

Animals and other research organisms

Policy information about studies involving animals; ARRIVE guidelines recommended for reporting animal research, and Sex and Gender in Research

Laboratory animals

All mice used in this study were maintained in the C57BL6/N genetic background. C57BL6/N mouse strains were obtained from the Charles River Laboratories (strain code: 027). Sct-/-, Sctr-/-, Sctfl/fl, and Sctrfl/fl mouse strains were previously described. Experiments in this work were carried out using 4-week-old to 20-week-old male or female mice.

Wild animals

There were no wild animals used in this study.

Reporting on sex

Both male and female mice were used in this study.

Field-collected samples

No field-collected samples were used in this study.

Ethics oversight

Animal care, welfare monitoring, experimental procedures, and euthanasia practices were carried out with the protocols approved by the Committee on the Use of Live Animals in Teaching and Research (CULATR) of the University of Hong Kong (protocol No.

5791-21). All animals were maintained in a facility accredited by the Association for the Assessment and Accreditation of Laboratory Animal Care International (AAALAC).

Note that full information on the approval of the study protocol must also be provided in the manuscript.

Plants

Seed stocks	N/A
Novel plant genotypes	N/A
Authentication	N/A
